# Modifying the Cyanobacterial Metabolism as a Key to Efficient Biopolymer Production in Photosynthetic Microorganisms

**DOI:** 10.3390/ijms21197204

**Published:** 2020-09-29

**Authors:** Maciej Ciebiada, Katarzyna Kubiak, Maurycy Daroch

**Affiliations:** 1 School of Environment and Energy, Peking University Shenzhen Graduate School, 2199 Lishui Rd., Shenzhen 518055, China; maciej.ciebiada@dokt.p.lodz.pl; 2 Institute of Molecular and Industrial Biotechnology, Lodz University of Technology, 4/40 Stefanowskiego Str, 90-924 Lodz, Poland

**Keywords:** cyanobacteria, polymers, metabolism, polyhydroxyalkanoates, cellulose, extracellular polymeric substances

## Abstract

Cyanobacteria are photoautotrophic bacteria commonly found in the natural environment. Due to the ecological benefits associated with the assimilation of carbon dioxide from the atmosphere and utilization of light energy, they are attractive hosts in a growing number of biotechnological processes. Biopolymer production is arguably one of the most critical areas where the transition from fossil-derived chemistry to renewable chemistry is needed. Cyanobacteria can produce several polymeric compounds with high applicability such as glycogen, polyhydroxyalkanoates, or extracellular polymeric substances. These important biopolymers are synthesized using precursors derived from central carbon metabolism, including the tricarboxylic acid cycle. Due to their unique metabolic properties, i.e., light harvesting and carbon fixation, the molecular and genetic aspects of polymer biosynthesis and their relationship with central carbon metabolism are somehow different from those found in heterotrophic microorganisms. A greater understanding of the processes involved in cyanobacterial metabolism is still required to produce these molecules more efficiently. This review presents the current state of the art in the engineering of cyanobacterial metabolism for the efficient production of these biopolymers.

## 1. Introduction

Cyanobacteria are single-celled or filamentous organisms belonging to the kingdom of Prokaryotes that are capable of oxygenic photosynthesis. Model strains such as freshwater *Synechococcus elongatus* PCC 7942 (*S. elongatus* PCC 7942), *Synechocystis* PCC 6803 (*S.* sp. PCC 6803), marine *Synechococcus* PCC 7002 (*S.* sp. PCC 7002), filamentous *Nostoc* sp. PCC 7120, and *Anabaena* sp. PCC 7120 [1,2] are the most studied representatives of cyanobacteria. In recent years there is an increased interest in developing other strains of cyanobacteria that are better suited to industrial applications thanks to their high growth rate [3] or high-temperature resistance [4,5]. In the process of autotrophic growth, they consume carbon dioxide, an important greenhouse gas, and contribute to reducing its level, which makes them an attractive host for biotechnological processes. Cyanobacteria utilize light as an energy source potentially resulting in relatively low financial outlays for their cultivation. However, ensuring uniform access to light for cyanobacterial cultures is still a challenge, and therefore the cultivation process in bioreactors is still being developed [6]. As a result of photosynthesis, the carbon is fixed and then can be converted into useful biofuels or biochemical substances such as biochemicals or biomaterials. There are many potential applications of cyanobacteria, such as wastewater treatment, production of fertilizers, vitamins, enzymes, pharmaceuticals, and hydrogen [1,7]. However, the widespread use of cyanobacteria in industrial settings requires basic research and development of new, technically applicable strains. Optimizing the conditions for reproduction of these bacteria requires a good understanding of the conditions under which they produce the expected product with the greatest efficiency, and often also modifying their metabolism. Examples of important issues related to the industrial use of cyanobacteria are insufficient growth rate and instability of modified strains [8,9]. The doubling time of cyanobacteria is about 7 h in natural conditions. More recently, strains capable of doubling in 2 h have been developed [8], which indicate that under favorable conditions, photosynthetic microorganisms are capable of growth rates similar to those of heterotrophs. Genetic instability of engineered strains may be overcome by e.g., reducing the natural rate of mutations by introduction of exogenous polymerases, better control of the ploidy of the cyanobacterial cell factory and ensuring full segregation across the population, and development of stringent expression control elements [9,10]. There are many metabolic pathways in cyanobacteria that could be exploited for the production of useful biochemicals from carbon dioxide. The main source of energy for the production of these compounds is photosynthesis, during which, ATP and reducing equivalents are produced that later empower the carbon assimilation and biosynthetic processes. Modifying and controlling the regulatory factors and pathways associated with the Calvin-Benson-Bassham cycle (CBB) can increase the efficiency of the photosynthesis process, and thus contribute to the more efficient synthesis of carbon-rich compounds. One of the pathways associated with CBB is the tricarboxylic acid (TCA) cycle. This cycle allows the cell to generate twelve high-energy bonds from one acetyl-CoA molecule, provides reducing equivalents and precursor compounds for the synthesis of valuable compounds such as polyhydroxyalkanoates, succinic acid, or ethylene.

Biological production of useful metabolites in cyanobacteria is both active and attractive research area [1,11]. However, due to growing demand for new materials synthesized in environmental-friendly processes, biopolymers seem to be particularly interesting, and intensively studied in cyanobacteria. In this paper, we focus on the production of biopolymers such as glycogen, PHB and extracellular polysaccharides, which are already successfully used in various industries and with the transformation of the world economy to less reliant of fossil carbon are expected to be even more prevalent in the near future. A thorough understanding of the influence of environmental conditions such as the availability of chemical elements, temperature, and lighting, as well as the regulation of gene expression and enzymatic activities, facilitates the creation of commercially viable strains. Some of these polymers can be already economically produced by heterotrophic microorganisms. Therefore the next step in their sustainable production is to transfer their biosynthesis into photosynthetic microorganisms such as cyanobacteria to directly connect the processes of carbon fixation and polymer synthesis without the need for employing an intermediary carbohydrate platform [1,12]. In the following parts of this article, an overview of the current state of the implementation and modification of carbon compounds metabolism will be presented. Then, research aiming at the development of potentially commercially profitable strains that produce glycogen, polyhydroxyalkanoates, and extracellular polymeric substances will be discussed.

## 2. Photosynthesis: Generating Energy for Biopolymer Synthesis

One of the essential features of cyanobacteria distinguishing them from most other bacteria groups is their ability to carry out photosynthesis. The process in cyanobacteria has two main phases. The light-dependent phase is responsible for the transformation of energy, during which light energy is absorbed, which is converted into energy of chemical bonds, and oxygen is released. During the light phase in cyanobacteria, light is collected by phycobilisomes whose subunits are encoded by core and antenna genes, characterized by decreased expression in the event of excess light. In the light-independent phase, it is CBB, i.e., the substance transformation phase, when the energy of the chemical bonds formed during the light phase, is used to synthesize organic compounds with the use of inorganic carbon. Light quanta during photosynthesis are assimilated by chlorophyll, which is found in reaction centers and the inner membrane complexes and bound to the binding protein, which forms the functional cores of photosystem I (PSI) and photosystem II (PSII) [13]. In *S.* sp. PCC 6803, around 80% of chlorophylls are bound to PSI [14,15]. A fundamental problem for the proper functioning of cells is protection against the formation of reactive oxygen species caused by the excess of light energy. During photosynthesis, the consumption of ATP and NADPH is related to the assimilation of carbon with CO_2_, which is relatively stable in cyanobacteria. However, their production depends on the availability of photons. When their intensity is too high, the resulting excessive reducing power can cause stress and cell damage. The cyclic electron transfer chain is a mechanism that partially solves this problem as it allows the production of ATP without forming NADPH. Another way is to regulate the ratio of photosystems. The *psbA* gene, which encodes the photosystem II D1 protein, is induced to increase the presence of PSII [16]. Also, for chlorophylls, if not bound or suppressed by carotenoids, reactive oxygen species can be formed, especially under unfavorable conditions such as excess light. For this reason, the expression of a number of genes related to chlorophyll biosynthesis, such as *chlB*, *chlL*, *chlN*, *chlP*, *hemA*, *hemF*, *ho1*, *por*, and *ycf59* (*S.* sp. PCC 6803), is reduced under conditions of too much exposure to light [16]. Of the key importance is also the decrease in the synthesis of 5-aminolevulinic acid, the precursor of tetrapyrroles, which form the active core of chlorophyll, through transcriptional and post-translational regulations [17,18]. Reducing the amount of chlorophyll is not the only way to defend against excess energy. To dissipate excess energy between unevenly excited photosystems, some of it can be transferred from one photosystem to another. This can be done via energy spillover between photosystems, or through state change, i.e., when the phycobilisome dissociates from PSII and attaches to PSI. These changes last short periods of time, i.e., from seconds to minutes [13]. Another photoprotecting process is non-photochemical quenching (NPQ), where excess energy in PSII is dissipated as thermal energy. Mutant *S.* sp. PCC 6803 deprived of orange carotenoid protein, on which this process depends in cyanobacteria, was more susceptible to damage caused by excess light than the wild type (WT) [19]. An important mechanism is also the degradation of the phycobilisome by the dimeric NblA protein, which marks this complex for the Clp protease. This mechanism also occurs under nitrogen starvation conditions [4,16,20].

**Figure 1:**
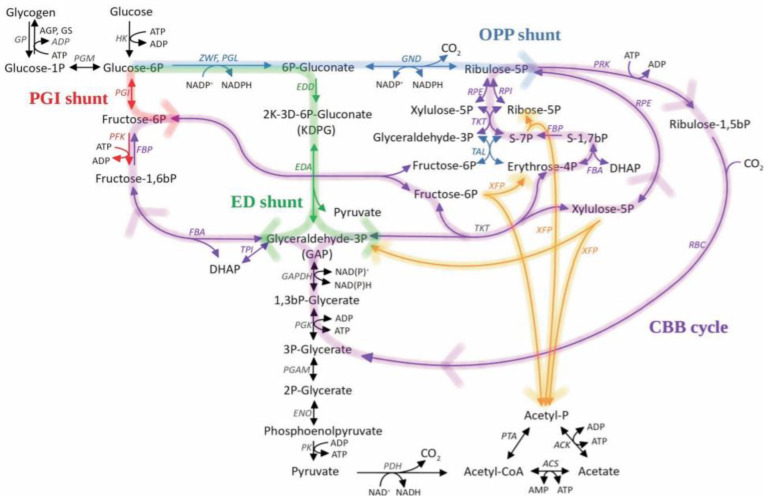
Relationship between the Calvin-Benson-Bassham (CBB) Cycle and Glycolytic Pathways in cyanobacteria. Abbreviations: acetate kinase (ACK), acetyl-CoA synthetase (ACS), glucose-1-phosphatase (AGP), KHG/KDPG aldolase (EDA), phosphogluconate dehydratase (EDD), enolase (ENO), fructose-bisphosphate aldolase (FBA), fructose bisphosphatase (FBP), glyceraldehyde 3-phosphate dehydrogenase (GAPDH), 6-phosphogluconate dehydrogenase (GND), glycogen phosphorylase (GP), glycogen synthetase (GS), hexokinase (HK), pyruvate dehydrogenase (PDH), phosphofructokinase 1 (PFK), phosphoglycerate mutase (PGAM), glucose-6-phosphate isomerase (PGI), phosphoglycerate kinase (PGK), 6-phosphogluconolactonase (PGL), phosphoglycerate mutase (PGM), pyruvate kinase (PK), phosphoribulokinase (PRK), phosphate acetyltransferase (PTA), ribulose-1,5-bis-p carboxylase/oxygenase (RBC), ribulose-phosphate 3-epimerase (RPE), ribose-5-phosphate isomerase (RPI), transaldolase (TAL), transketolase (TKT), triose-phosphate isomerase (TPI), xylulose-5-phosphate/fructose-6-phosphate phosphoketolase (XFP), glucose-6-phosphate 1-dehydrogenase (ZWF).Violet is the Calvin-Benson Cycle; Green is the Entner-Doudoroff shunt for CBB; Blue is the Oxidative Pentose Phosphate shunt for CBB; Red is PGI shunt for CBB; Orange is the hypothetical model of phosphoketolase integration central carbohydrate metabolism. Reproduced with permission from Makowka A. et al., Glycolytic Shunts Replenish the Calvin–Benson–Bassham Cycle as Anaplerotic Reactions in Cyanobacteria; published by Molecular Plant, 2020.

Carbon dioxide is the primary source of carbon for cyanobacteria, which makes it crucial both for their metabolism and their use as cell factories. This photosynthetic phase consists of three steps: carboxylation, reduction, and regeneration. CO_2_ is fixed by cyanobacteria by carboxylation of ribulose-1,5-bisphosphate in the CCB cycle. Balancing the appropriate enzyme activity in the central carbon metabolism enzymes that are connected with the CBB by, for example, overexpression of one of four separate enzymes (fructose bisphosphate aldolase, sedoheptulose bisphosphatase, RuBisCO, or transketolase) favored an increase in the carbon binding capacity in *S.* sp. PCC 6803 [21]. Modification of the expression of these CBB-related enzymes increased the rate of heterologous ethanol production by *S.* sp. PCC 6803 [22]. CCB in cyanobacterial cells is coordinated by the association and dissociation of the Phosphoribulokinase/CP12/Glyceraldehyde-3-phosphate dehydrogenase (PRK/CP12/GAPDH) complex. Creation of that complex results in the inactivation of the cycle in the absence of light. This complex dissociates by exchanging NADP^+^ for NAD^+^. For the mutant cyanobacteria of *S. elongatus* PCC 7942 in which the *cp12* gene was disrupted (ScΔCP12), the decrease in oxygen consumption during the dark photoperiod was observed. There was also a 2.5-fold increase in ribulose 1,5-bisphosphate and a 50% decrease in fructose 6-phosphate content in mutant strains under dark photoperiod conditions, which was associated with an increase in PRK protein activity. However, the CP12 deletion strain cultured in 12L:12D photoperiod, exhibited growth inhibition when compared to WT cells [23]. It was observed that because of the existence of glycolytic shunts, the glycolytic pathways have a crucial effect on CCB in cyanobacteria (Figure 1). Through a series of mutant strains of *S.* sp. PCC 6803, it was shown that cyanobacteria with disturbed glycolytic pathways have impaired growth relative to the WT. Strain Δ*eda (sll0107::*em^R^*)* with impaired Entner-Doudoroff (ED) pathway exhibited 18% growth retardation with respect to the WT. An identical result of 18% of growth depletion was obtained for the Δ*zwf (slr1843::*cm^R^*)* mutant with impaired oxidative pentose phosphate (OPP) pathway. For the Δ*eda* Δ*pfk (sll0107::*gm^R^, *sll1196::*km^R^, *sll0745::*sp^R^*)* mutant, which had impaired Embden-Meyerhof-Parnas (EMP) and ED pathways, the growth was reduced by 37% compared to WT. In contrast, for the strain Δ*eda* Δ*gnd (sll0107::*gm^R^, *sll0329::*em^R^*)*, which had impaired OPP and ED pathways, the growth was reduced by 60%. Simultaneously the mutant Δ*eda* exhibited 145% increased glycogen levels. For all these mutants, except for Δ*pfk (sll1196::*km^R^, *sll0745::*sp^R^*)*, a decrease in glycogen degradation was observed, the strongest for the mutants Δ*zwf*, Δ*gnd*. Moreover, in the mutants, Δ*zwf*, Δ*gnd (sll0329::*em^R^), as well as Δ*eda*, a delay in the initiation of the CBB pathway was observed compared to WT. That was evidenced by a decrease in photochemical fluorescence quenching of the system in mutant strains. This was because the pathways are involved in providing extra carbohydrates to complete the CBB cycle. The most significant decrease in photochemical quenching was observed for the Δ*eda* mutant and the smallest for Δ*zwf* [1,24]. Another method of modifying the CBB cycle is the infection of *Prochlorococcus* and *Synechococcus* cultures with cyanophages encoding the CP12 protein. It was shown that for infected cells, the NADPH/NADP ratio increased two-fold, which was associated with a decrease in the activity of the CBB cycle and enhancement of the OPP pathway [25].

Based on the above-mentioned studies, it can be concluded that the processes of photosynthesis and the pathways in which biopolymers are formed, are closely related. The analysis and optimization of these relationships are crucial for the development of a commercially optimal cyanobacterial culture. Important advances in the understanding of molecular machinery responsible for circadian cycle regulation in cyanobacterial cell done in the above-cited studies are still insufficient to gain full control over efficient autotrophic production of biopolymer of choice. Some most significant progress has been made thanks to the studies aimed at understanding the regulatory pathways responsible for light protection [14,17]. If these fields of research are explored further, one can expect some new cyanobacterial strains engineered in the direction of increased “photoresistance” in the upcoming years. Autotrophism of a cyanobacterial cell impacts other metabolic pathways that are well studied in heterotrophic bacteria. Photosynthetic production of the central metabolites is closely connected to central carbon metabolism pathways (such as EMP, ED, OPP) and fuels the TCA cycle, which in consequence, play different roles in cyanobacterial system than in heterotrophic bacteria. This sometimes may cause misleading data interpretation, and one should be aware of these major differences when constructing the strain.

## 3. Unusual TCA Cycle as a Source of Key Biopolymer Intermediates

**Figure 2:**
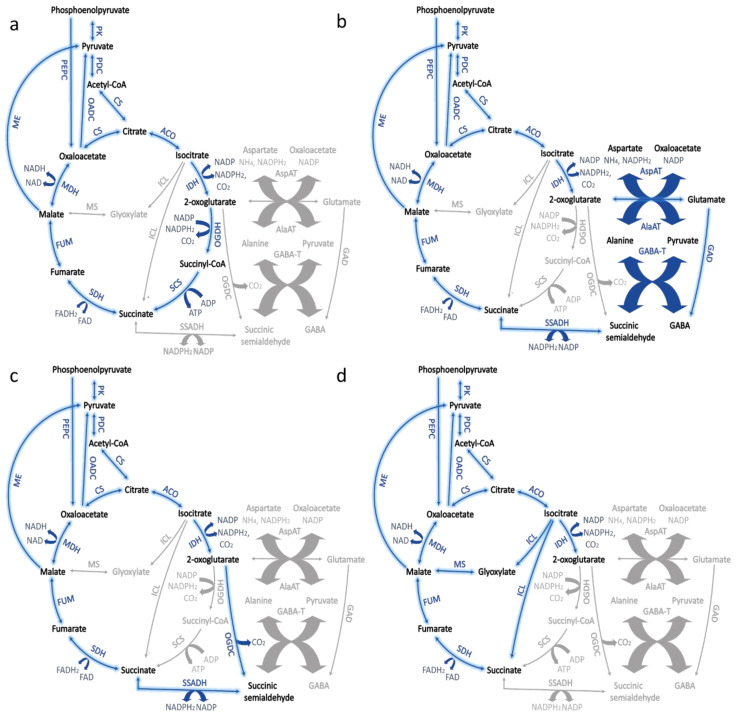
Modified tricarboxylic acid (TCA) pathway in cyanobacteria. (**a**) Typical bacterial TCA cycle; (**b**) Bypass of the TCA with GABA shunt; (**c**) Pathway converting 2-oxoglutarate to succinic semialdehyde; (**d**) Bypass of the TCA with glyoxylate shunt. Abbreviations: aconitase (ACO), alanine aminotransferase (AlaAT), aspartate aminotransferase (AspAT), citrate synthase (CS), fumarase (FUM), GABA aminotransferase (GABA-T), glutamate decarboxylase (GAD), isocitrate lyase (ICL), isocitrate dehydrogenase (IDH), malate dehydrogenase (MDH), malic enzyme (ME), malate synthase (MS), oxaloacetate decarboxylase (OADC), 2-oxoglutarate decarboxylase (OGDC), 2-oxoglutarate dehydrogenase complex (OGDH), pyruvate dehydrogenase complex (PDC), phosphoenolpyruvate carboxylase (PEPC), pyruvate kinase (PK), succinyl CoA ligase (SCS), succinate dehydrogenase (SDH), succinic semialdehyde dehydrogenase (SSADH).

In cyanobacteria, the tricarboxylic acid cycle (TCA), has a significant impact on the production of biopolymers. Since the majority of cellular energy is generated in the process of photosynthesis, its function is somehow different to one in heterotrophic organisms. The cycle supplies the essential precursors for many biopolymers such as polyhydroxyalkanoate, or by indirect influence on synthesis through interactions with other metabolic pathways, as is the case of glycogen or extracellular polysaccharides. The Krebs cycle in cyanobacteria proceeds in an unconventional manner when compared to heterotrophic organisms. There is no 2-oxoglutarate dehydrogenase (OGDH) encoded in cyanobacterial genomes [26]. Due to the impact of the TCA cycle on the production of energy intermediates in aerobic respiratory organisms, researchers have attempted to determine how the lack of this enzyme is compensated for in photosynthetic cyanobacteria. Several metabolic pathways exist to make up for this enzyme, and they rely on the shunt pathways involved in the metabolism of γ-aminobutyric acid (GABA), succinic semialdehyde, or glyoxylate (Figure 2) [27,28]. *S.* sp. PCC 7002 produces 2-OG decarboxylase and succinic semialdehyde dehydrogenase in place of succinyl-CoA ligase and 2-oxoglutarate dehydrogenase (Figure 2c) [29]. *S.* sp. PCC 6803 uses GABA shunt to compensate for the lack of these enzymes (Figure 2b) [30,31]. Another variant of TCA in cyanobacteria depends on the glyoxylate shut and is present in several strains of cyanobacteria, including *Chlorogloeopsis fritschii* PCC 9212 (Figure 2d) [32]. Importantly, it has also been shown that having other enzymes: fumarase, malate dehydrogenase, and succinate dehydrogenase is not necessary under both day and night conditions. For this purpose, Δ*fum*, Δ*me2*, and Δ*sucd* mutants were created that lost the function of these enzymes. These cyanobacteria were grown under constant lighting conditions or alternating 12 h day-night cycles. Their growth was monitored and compared to the wild-type strain. The colony area was measured, and it was shown that although the size of the mutants’ colony was smaller than that of the WT, the viability of the mutants was stable, and the growth rates of both strains were similar [28]. For cyanobacteria, the primary function of the TCA cycle is in the production of precursor metabolites associated with oxaloacetate and 2-oxoglutarate, which is needed in the process of nitrogen assimilation. Energy production in *S. elongatus* PCC 7942 is also associated with the OPP, which during glycogen oxidation during the dark phase can create 24.4 mol ATP / mol glucose [28]. However, whether the metabolism of compounds associated with the TCA pathway will be more strongly associated with the OPP pathway or more focused on glycogen polymerization depends on the availability of nitrogen, in the case of nitrogen starvation, a larger flux of carbon will be involved in the synthesis of ADP-glucose, which is a precursor of glycogen in most cyanobacteria [33]. In thermophilic *Thermosynechococcus* strains, the unusual substrate specificity of sucrose synthase and its preference towards ADP-glucose over UDP-glucose indicates a closer connection between glycogen and sucrose in extremophilic cyanobacteria [34]. Another function of the TCA pathway is the recovery of fumarate as a product of the synthesis of arginine and nucleotides; however, *S. elongatus* PCC 7942 can excrete fumarate into the medium [35]. Whereas in *S.* sp. PCC 6803 it has been shown that the stream of metabolites from 2-oxoglutarate to succinate is weaker than in a typical TCA as it is bypassed by a GABA shunt (Figure 2b) [27,28,36]. The pathway referred here as typical TCA is presented in Figure 2a and relies on the presence of citrate synthase, aconitase, isocitrate dehydrogenase, 2-oxoglutarate dehydrogenase complex, succinyl CoA ligase, succinate dehydrogenase, fumarase, and malate dehydrogenase [36].

To sum up, advances in the understanding of the cyanobacterial metabolism revealed that carbon-fixing Calvin-Benson-Bassham, and TCA cycles are fundamentally connected to the biosynthesis of biopolymers. Notably, the CBB in cyanobacteria has a leading role in energy generation; therefore, TCA is less critical for maintaining the cellular energy balance than it is in heterotrophic systems. The exciting consequence of this fact is that a potentially more explicit genetic interference in this pathway can be tolerated by cyanobacteria when compared with their heterotrophic counterparts [26]. Another vital aspect of TCA variations observed in different cyanobacterial strains is the resulting diversity of metabolites available for the bioprocesses to be engineered. Having less of an impact on energy supply, the TCA cycle in cyanobacteria acts, primarily, as a source of intermediates for biosynthesis of, among others, reserve material (like polyhydroxyalkanoates). Taking into consideration ever-increasing abundance of genomic information of new cyanobacterial strains, TCA cycle variability should be at the forefront of the description in the newly described strains to evaluate its potential applicability.

## 4. Glycogen and Carbon Storage in Cyanobacteria

**Figure 3:**
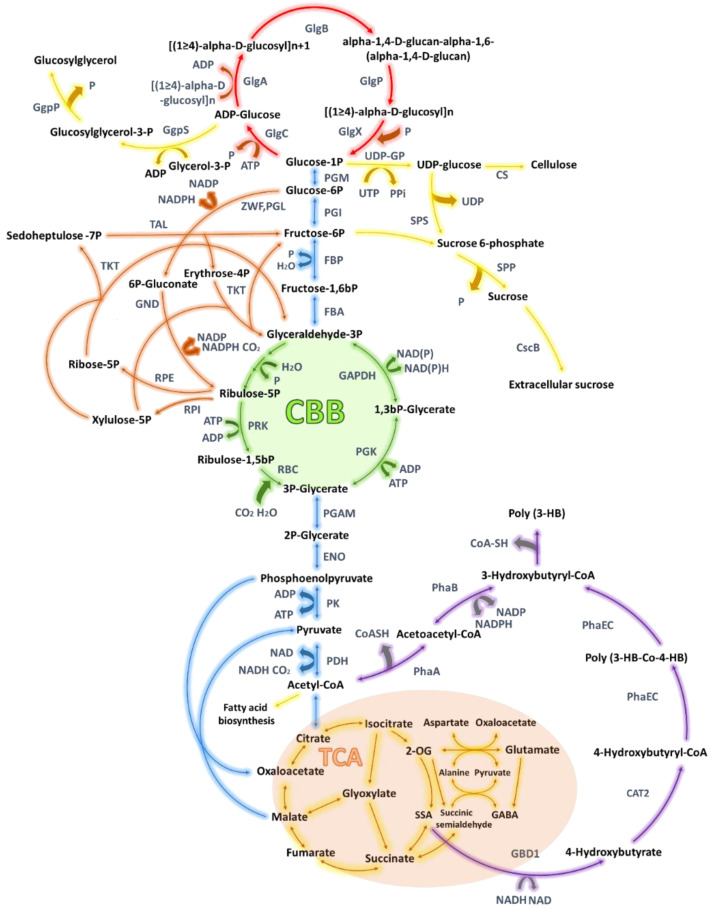
Impact of central carbon metabolism pathways in cyanobacteria on biopolymers production. Tricarboxylic acid cycle is gold, polyhydroxybutyrate (PHB) synthesis pathway is purple, Calvin-Benson- Bassham cycle is green, oxidative pentose phosphate is orange, non-CBB glycolysis pathway is blue, glycogen metabolism is red, other pathways are yellow. Abbreviations: 4-hydroxybutyryl-CoA transferase (CAT2), cellulose synthase (CS), sucrose transporte (CsbB), enolase (ENO), fructose-bisphosphate aldolase(FBA), fructose bisphosphatase (FBP), glyceraldehyde 3-phosphate dehydrogenase (GAPDH), 4-hydroxybutyrate dehydrogenase (GBD1), glucosylglycerol-phosphate phosphatase (GgpP), glucosylglycerol-phosphate synthase (GgpS), glycogen synthase (GlgA), 1,4-alpha-glucan branching enzyme (GlgB), glucose-1-phosphate adenylyltransfe (GlgC), glycogen phosphorylase (GlgP), glycogen debranching enzyme (GlgX), 6-phosphogluconate dehydrogenase (GND), pyruvate dehydrogenase (PDH), phosphoglycerate mutase (PGAM), glucose-6-phosphate isomerase (PGI), phosphoglycerate kinase (PGK), 6-phosphogluconolactonase (PGL), phosphoglycerate mutase (PGM), acetyl-CoA acetyltransferase (PhaA), acetoacetyl-CoA reductase (PhaB), poly(R)-hydroxy alkanoic acid synthase (PhaEC), pyruvate kinase (PK), phosphoribulokinase (PRK), ribulose- 1,5-bis-p carboxylase/oxygenase (RBC), ribulose-phosphate 3-epimerase (RPE), ribose-5-phosphate isomerase (RPI), sucrose-phosphate phosphatase (SPP), sucrose-phosphate synthase (SPS), transaldolase (TAL), transketolase (TKT), glucose-1-phosphate uridylyltransferase (UDP-GP), glucose-6-phosphate 1-dehydrogenase (ZWF).

TCA cycle is the primary source of precursor molecules for cyanobacteria. Figure 3 presents the relationship between central carbon metabolism pathways concerning the biosynthesis of some important polymers and their building blocks. An example of such compound is glycogen. Glycogen is a branched α-polyglucan, playing the role of a cellular carbon reservoir. Its metabolism has a key impact on bacterial cell adaptation to various environmental conditions and its growth. Over the past decades, it has been possible to characterize the metabolic pathways associated with the synthesis and catabolism of this polymer [37,38]. Numerous studies are underway in which these pathways undergo genetic manipulation to discover the effects of this polymer on the survival and functioning of cyanobacteria. One of those modifications is the disruption or complete removal of glycogen synthesis. This is usually caused by the desire to change the carbon flow in the cell to increase the production of other metabolites. It was shown, however, that reduced levels of glycogen often results in significant impairment in cell homeostasis and finally, the availability of carbon for other metabolites is limited too [1]. 

Glycogen synthesis begins with the transformation of the glucose-1-phosphate precursor by ADP-glucose pyrophosphorylase (GlgC), resulting in the formation of ADP-glucose. Subsequently, glycogen synthase (GlgA), polymerizes these monomers forming α-1,4-glycosidic bonds. The linear chains of polyglucose are branched through α-1,6-glycosidic bonds with the GlgB enzyme (Figure 3) [39]. In some cyanobacteria, including the *S.* sp. PCC 6803, there are two isoforms of GlgA proteins. The first isoform participates in the synthesis of glycogen, creating mainly medium-length chains (8-18), while the second isoform mainly participates in the synthesis of longer and shorter chains. In S. sp. PCC 6803 mutants incapable of producing one isoform, the compensation effect from the other isoform was observed [27,40].

To understand the effect of these enzymes on cell function, knock-outs and knock-downs of the *glgC* or *glgA* genes have been constructed to block or limit glycogen synthesis in the cell. The first study resulting in inhibition of glycogen production was carried out in *S.* sp. PCC 6803 deletion mutant strain, in which the gene encoding GlgC was removed [41]. Inactivation of two copies of the gene encoding GlgA protein has also been shown to have a similar effect, with the elimination of only one copy of this gene having little effect on the *S.* sp. PCC 6803 strain. In addition, mutant strain with no-GlgA suffered from impairments of the growth and phycobilisome degradation capacity [42]. However, for the *S.* sp. PCC 7002 strain, the effect of analogous elimination of a single gene has already caused a 33–40% decrease in glycogen content [43]. Small amounts of glycogen have been reported in *S.* sp. PCC 7002 strains with GlgA and GlgC proteins inhibition. These observations suggest that some other, yet-to-be-identified enzymes may be involved in glycogen production [44]. An example of a method aimed at limiting glycogen metabolism is the use of interference CRISPR-Cas9. In *S.* sp. PCC 6803 and *S. elongatus* PCC 7942 strains, induced dCas9, and sgRNA-*glgC* gene expression was used, which reduced the transcript level by 90% and accumulated only 25% of glycogen content typical of the *S.* sp. PCC 6803 WT strain. An analogous study using *S. elongatus* PCC 7942 revealed 73.4–93.8% lower transcript levels and glycogen accumulation at the level of 4.8% to 25.5% of the WT. Interestingly, the strain responded with increased biosynthesis of succinate [45,46]. Another solution was to use a tool based on the exogenous Hfq chaperone and the MicC scaffold (Hfq-MicC) to suppress the gene coding for the GlgC protein. It caused mutant *S.* sp. PCC 6803 decrease in expression of that gene by 75% [47]. Theophylline-responsive riboswitch has also been used, this resulted in a 90% decrease in glycogen accumulation in *S.* sp. PCC 6803 in the absence of theophylline, and the addition of 1100 µM theophylline increased glycogen accumulation by 300% in *S. elongatus* PCC 7942 [48,49]. The effects of disturbances in the glycogen synthesis pathway on cell growth depend on temperature and have practical importance for strain culturing. With respect to the WT, the mutant strain Δ*glgAI*-Δ*glgAII* of *S.* sp. PCC 7002 exhibited growth retardation at 38 °C, while at 30 °C growth rate was not changed [43,50]. Interestingly, *S.* sp. PCC 7002, in which the *glgA* genes had been deleted, was found to secrete about 30% more sugars than the WT after increasing salinity. Whereas, for the Δ*glgA* mutant of *S. elongatus* PCC 7942, glycogen level was lower by more than 90% in conditions of increased salt content. Meanwhile, for mutants Δ*glgC*, a decrease in tolerance to high salinity was noted. Interestingly, the inhibition of the gene encoding the GlgC protein in *S. elongatus* PCC 7942 strain resulted in a reduction of GlgA protein activity by 80%. However, for the same strain, the deletion of the *glgA* gene caused an increase in the activity of the GlgC protein by half [39,43,51]. Examples of studies with mutant cyanobacterial strains on glycogen are gathered in Table 1.

An example of research that creates a mutant that produces glycogen with greater efficiency than the wild type was the study in which the expression of *glgC* gene of *S. elongatus* PCC 7942 was increased. The engineered strain, where the *glgC* gene was under the control of the strong cpcB1 promoter, exhibited a simultaneous increase of sucrose biosynthesis, whilst its glycogen content was increased by 30–50% [48].

Changes in the level of expression of genes encoding proteins associated with the glycogen synthesis pathway also have a major impact on cell survival. The glycogen biosynthesis prediction in *S.* sp. PCC *6803*’s, using the CycleSyn model, indicated that the increase to the highest glycogen level in the cell occurs just before the end of the light photoperiod, followed by a decrease [60]. An important issue is, however, disturbance in the process of photosynthesis during the transition from the dark photoperiod to daylight. During the final hours of the dark photoperiod, the rate of glycogen degradation increases, most likely to provide the energy needed to initiate photosynthesis. Hellweger et al. performed a simulation for *Synechococcus*, suggesting that the *kaiABC* clock genes control the level of glycogen synthesis during the day [61,62]. Recently, using the mutated strain of *S. elongatus* PCC 7942 ∆*glgC*, it was shown that glycogen undergoes catabolism to support the pentose phosphate oxidative pathway, which is necessary to initiate photosynthesis during the beginning of light photoperiod. At the same time, this process modulates NADPH levels, which are necessary for photosynthesis, which is also confirmed by other studies [55,63]. These data coincide with the results of differential RNA sequencing (dRNA-seq), which showed that in *S. elongatus* UTEX 2973, a close relative of PCC 7942, an increase in the transcription of genes for glycogen catabolism during the dark phase of the photoperiod, and a decrease in the expression of the *glgC* and *glgA* genes, with simultaneous continuous oxidation of glucose from glycogen via pathways EMP or OPP [64]. Other studies also showed that glycogen is needed for proper cell function during the light phase of the photoperiod. The knock-out of the gene encoding the GlgC protein in the *S.* sp. PCC 6803 strain resulted in a 25% reduction in oxygen evolution during the light phase of the photoperiod [41]. Simultaneously, in the *S. elongatus* PCC 7942 strain; the oxygen evolution decreased by 50% for Δ*glgA* and Δ*glgC* mutants. However, the level of Chl did not change, and the phycocyanin concentration increased with respect to the WT. This was probably due to the low efficiency of energy transfer to the reaction center from pigments [51].

One of the most important osmoprotective compounds for cyanobacteria is glucosylglycerol, which requires ADP-glucose, i.e., the product of the enzyme GlgC, for its biosynthesis. The lack of this enzyme cannot be compensated by the increased synthesis of other compounds like sucrose, since they do not provide sufficient osmoprotection, which results in impaired cell function. However, also in the case of mutations in the *glgAI* and *glgAII* genes that led to an increase in glucosylglycerol and sucrose biosynthesis, oxygen secretion rates decreased and the doubling time increased by 15% [41,43,44,51]. In the case of nitrogen starvation, cyanobacteria can undergo the bleaching process; however, the mutant strain *S.* sp. PCC 6803, which lost its ability to synthesize glycogen, retained its green color, even though the wild strain showed signs of bleaching. However, unlike the wild type, the mutant growth rate was strongly reduced [42]. It was also established that during nitrogen deficiency, 6S RNA, which is a global transcription riboregulator, plays an important role. In the mutant Δ*ssaA* (6S RNA deletion) *S.* sp. PCC 6803, delays in adaptation to changes in nitrogen levels, due to, among others, a decrease in glycogen breakdown has been reported [65].

Lowering the glycogen content in the cell is also achieved by engineering its catabolism. Glycogen degradation is catalyzed by GlgX proteins, which hydrolyze α-1,6-glycosidic bonds, and GlgP glycogen phosphorylase, which hydrolyzes the α-1,4-glycosidic terminal bonds, thereby forming glucose monophosphate [39]. In *S.* sp. PCC 6803, this catabolism is controlled by two GlgX isoforms (GlgX1 and GlgX2) and two GlgP isoforms (GlgP1 and GlgP2) [27]. The gene encoding the GlgP protein was overexpressed in *Escherichia coli*, resulting in a decrease in glycogen content [66]. This may suggest that increasing the level of this protein can cause a decrease in the amount of glycogen in cyanobacteria. Another strategy is to reduce or eliminate the production of this enzyme to achieve the opposite effect. In one study in the *S.* sp. PCC 6803 strain, a mutant lacking GlgP was created that led to 420% of WT glycogen content in biomass [58]. Knock out of the *glgX* gene in the *Cyanothece* 51142 strain cyanobacterium resulted in faster growth and a higher rate of nitrogen fixation [67]. DNA methylation has recently been shown to influence the activity of genes associated with glycogen catabolism. Based on the study on *S.* sp. PCC 6803 mutants with inactivated genes of type I restriction methylation system which showed increased activity of GlgP, methylation of DNA may be a regulatory pathway controlling activity of its gene [68]. Interestingly in the study of Li and co-workers from 2014, in mutated strains of *S. elongatus* PCC 7942 with glycogen deficiency, the biosynthesis of a non-native product, isobutanol, served as an alternative carbon “sink”, boosting cell growth. This suggests that when optimizing the production of other carbon storage compounds, the similar effect could be achieved [69].

Glycogen, being important metabolite for keeping cellular homeostasis should not be treated as a cellular “cargo” which can be eliminated from the engineered strains. Numerous inefficient efforts to increase the metabolic flux towards the product of choice production via elimination of glycogen synthesis stands for this conclusion. In cyanobacteria, carbon fixation seems to be closely related to pathways gathering storage compounds. Therefore, redirecting glycogen carbon flux into other desirable products has led to insufficient productivity of the engineered strains. More systemic approaches, aiming at cellular homeostasis protection, should compile desired product production with both circadian clock regulatory machinery and glycogen storage pathways.

## 5. Production of PHA in Cyanobacteria

Another important polymer that is influenced by central carbon metabolism is polyhydroxyalkanoate (PHA). The ability to conduct sugar catabolism effectively affects the availability of PHA precursors [70]. PHA is a class of compounds consisting of hydroxy acid units. Biosynthesis of PHA occurs using PHA synthases, which determine what type of polymer will be synthesized by catalyzing the enantioselective polymerization reaction of (N)-hydroxyacyl-CoA thioesters to polyesters, where N is an alkyl or functional group. The most commonly produced PHA polymer in cyanobacteria is polyhydroxybutyrate (PHB). Its synthesis consumes acetyl-CoA, which is converted by PhaA to acetoacetyl-CoA. This compound is transformed into 3-hydroxy-butyryl-CoA, which is polymerized by PhaEC to PHB with the help of PhaB and with the participation of NADPH [71]. Depending on the size of the polymer, there are three classes of Short-Chain Length-PHA (SCL-PHA), consisting of 3 to 5 units, Medium Chain Length-PHA (MCL-PHA), consisting of 6 to 14 units, and Long-Chain-Length-PHA (LCL-PHA) consisting of more than 15 units. An important protein is PhaP, which is involved in controlling the upper size of PHB granules [72,73]. *S. elongatus* PCC 7942. in which the PHB operon gene from *R. eutropha* was inserted, was capable of a PHB yield of 25% dry matter [74]. Table 2 presents examples of studies on mutant strains of cyanobacteria producing PHB.

Methods that are used to increase the amount of PHB produced by cyanobacteria are, among others, those based on the use of dicyclohexylcarbodiimide metabolic inhibitors, 1,1-dimethyl urea, and carbonyl cyanide-m-chlorophenyl hydrazone. Their use in *S.* sp. PCC 6803 affected the NADPH/NADP^+^ ratio, which contributed to the increase in PHB production. However, the effect of this ratio on PHB production was characteristic of impaired metabolism conditions, such as nitrogen starvation. Also, the C:N ratio can significantly affect the amount of PHB produced [73,81,82,83]. PHB production efficiency is also affected by phosphorus and sulfur starvation, as well as the addition of other carbon sources. In the study of Hirai and co-workers, it was shown that sulfur starvation resulted in the production of PHB in *S.* sp. PCC 6803 at 0.9% dry cell weight (DCW), and for N starvation conditions this level was 3% DCW. In the case of phosphorus starvation, accumulation only occurred after prolonged exposure to starvation and amounted to 2%. For *Scytonema geitleri*, the highest PHB production of 7.12% was obtained under acetate supplementation [77,83].

It has been shown that glycogen availability is important under nitrogen starvation, especially for the mutant strains *S.* sp. PCC 6803 with the *glgP2* knock-out [71]. During nitrogen starvation, in the case of mutant *S.* sp. PCC 6803 strains lacking both Pfk isoforms participating in the glycolysis pathway (EMP), lower PHB production than in the wild type was observed under both the continuous light phase of the photoperiod and the alternating light and dark phases of the photoperiod. For mutants with impaired OPP pathway by *gnd* gene knock-out, the PHB level was slightly higher than the *phk* mutant but lower than the WT, while for the mutant that does not produce Eda, from the Entner-Doudoroff (ED) pathway, the production of PHB was not impaired [71]. Using the mutant Δ *phaEC S.* sp. PCC 6803, it was proven that the production of PHB did not affect the growth rate of cyanobacteria, also under nitrogen starvation [77]. Overexpression of the gene encoding FabG protein increases PHB production by redirecting carbon from fatty acids towards PHB synthesis [78]. Regulators that control catabolic processes in cyanobacteria, such as SigE RNA polymerase, sigma factor, and Rre37 response regulator also have an effect on PHA biosynthesis. Strains overexpressing each of these genes have shown improved accumulation of PHA in *S.* sp. PCC 6803, especially during nitrogen starvation. The reason for this was that these regulators increase the metabolic flow from glycogen (and, in the case of SigE, also from TCA and pentose phosphate pathways) to PHB. It was also shown that for *S.* sp. PCC 6803 strains with double overexpression of *rre37* and *sigE* genes, the transcription level of *phaA* and *phaB* genes increased 4-fold, while *phaC* and *phaD* genes increased 3-fold [70,84,85,86]. In another study, disruption of PHB synthesis in Δ*GCAX-*Δ*BK* strains, knocking out the PHB pathway and overexpressing the *glgC*/*glgA* genes, increased the glycogen content from 25% for WT to 54% after addition of 3 mM H_2_O_2_ to the mutant culture [87]. The addition of 0.4% acetate to the culture medium of *S.* sp. PCC 6803 mutant overexpressing the *phaAB* genes, resulted in an increase of accumulated PHB from 26% to 35% under nitrogen starvation conditions [75]. It has also recently been shown that in *S.* sp. PCC 6803, there exists the Slr0058 protein that belongs to the transcription unit TU2718. This protein is similar to the PhaF protein from *Pseudomonas putida*, which is a regulatory phytase involved in PHB metabolism, but it does not contain any DNA binding domain. The lack of the Slr0058 protein has been shown to result in a weakening of growth with a simultaneous increase in PHB production [88]. Also, another protein from the TU2718 unit: Slr0060 may be involved in PHB depolymerization, as the mutant that did not produce the Slr0060 protein produced more PHB, and no significant difference in growth rate versus WT was observed. Under conditions of alternating light and dark phases of photoperiod, the PHB level increased [88].

Understanding the role of polyhydroxyalkanoates and their relationship with other central cyanobacterial metabolic pathways is far from complete. On the one hand, there are apparent interrelations between these pathways, especially glycogen biosynthesis and breakdown; on the other hand, complete elimination of the PHB production pathway does not seem to affect the cellular fitness [76]. The complexity of this relationship, combined with the need for developing sustainable alternatives to traditional plastics, is likely to drive further research in the efficient production of polyhydroxyalkanoates in cyanobacteria. For the time being, the approaches based on utilization of genetic elements from unrelated organisms e.g., *Ralstonia euthropha* and synthetic gene circuits is likely to provide high, albeit batch, productivities of these biopolymers in cyanobacteria.

## 6. EPS Produced by Cyanobacteria

**Figure 4:**
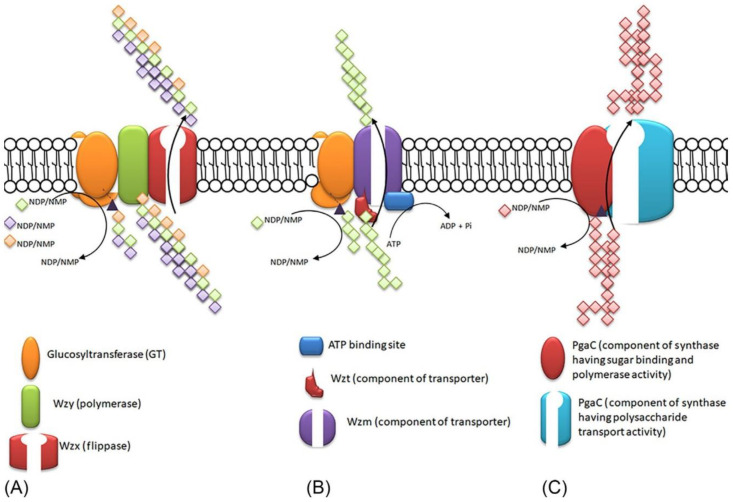
Scheme of the pathways of extracellular polymeric substances (EPS) formation. (**A**) Wzx-Wzy pathway, (**B**) ABC-transporter pathway, (**C**) synthase-dependent pathway. Reproduced with permission from Singh S., Cyanobacteria; published by Academic Press, 2019.

Cyanobacteria produce outer cell membrane that combines the characteristics of both gram-negative and gram-positive bacteria [89]. Arguably the most biotechnologically important components of these structures are extracellular polymeric substances (EPS), i.e., natural polymers, which, unlike released polymer substances (RPS), are permanently attached to the outer layers of the cell. The polymeric substances bound to the cell surface may have the following forms: capsules, sheaths, and slime. Among natural functions of EPS there are protection against environmental factors, such as drying or UV radiation, colonies creation, and spreading on surfaces [90]. The EPS produced by cyanobacteria are more complex than those produced by other types of bacteria. They usually contain from 6 to 13 different monosaccharides, as well as ester, acetyl sulfate, and peptide groups [89]. They are potentially applicable in e.g., the bioremediation of environments contaminated with heavy metals or reclamation of desert soils. EPS produced by cyanobacteria has been shown to increase soil moisture [91,92]. Production of EPS in a cyanobacterial cell is complex. Typically, most of the enzymatic steps in exopolysaccharide precursor biosynthesis occur inside the cell, while polymerization and secretion are localized in the cell envelope. However, there are also examples of extracellularly synthesized polysaccharides such as, e.g., dextran or levan. Their biosynthesis is mediated by glycosyltransferases (GT), which are secreted and covalently bound to the cell surface [93,94]. There are three main routes to EPS formation [95], presented in Figure 4.

In cyanobacteria genes involved in the biosynthesis of polysaccharides are concentrated in smaller units or are dispersed in the genome, a feature unusual for most other EPS-producing bacteria [96]. A positive relationship has been demonstrated between genome size, distribution, and number of protein-coding genes involved in EPS production. This is likely due to gene duplication events during evolution. However, this relationship is much less visible when the distribution and number of genes are analyzed in terms of cell morphology and type of habitat. Interestingly, unlike most other cyanobacterial strains for unicellular strains of *Synechococcus* and *Prochlorococcus*, it has been shown that they have lost most of the proteins associated with EPS production during adaptation to the marine environment [89]. EPS is produced by adding monosaccharides to the polymer chain. Monomers are obtained by cleaving from di- or tri-saccharides, with many specialized enzymes involved [95,97,98,99]. Brief description of each of the three mechanisms of secretion and polymerization of EPS in cyanobacteria is presented in Figure 4.

The first of the EPS polymerization mechanism is dependent on the Wzx/Wzy proteins [95]. Most of the polysaccharides produced by this pathway are heteropolymers. Individual monosaccharide units are linked to the undecaprenyl diphosphate (C55-UPP) on the cytosol side of the inner membrane and are assembled by the GT group. Then they are moved through the cytoplasmic membrane utilizing the so-called flippase Wzx protein. In the next stage, their polymerization takes place in the periplasm catalyzed by the Wzy protein, and then they are exported to the cell surface [100,101,102]. This transport depends on polysaccharide copolymerase (PCP) and extra-membrane polysaccharides (OPX) [101,102]. Other complexes of proteins involved in the described mechanism are Wza-Wzc, and most likely Wzz and Wzb proteins. They are probably involved in EPS export, the polymerization process, or the control of chain length and number of monomer copies. An example of a polymer synthesized by this pathway is lipopolysaccharide (LPS), which has an unusual structure due to lack of heptose and 3-deoxy-d-manno-octulosonic acid, while lipid A is phosphate-free and contains odd-chain hydroxylated fatty acids. Marine cyanobacterial LPS particles in marine strains *Synechococcus* WH8102 and CC9311 LPS lack heptose and 3-deoxy-d-manno-octulosonic acid while it contains 4-linked glucose as well as glucosamine and galacturonic acid [103]. The lipid A region is phosphate-free in these strains, but has a single galacturonic acid and odd-chain hydroxylated fatty acids [103]. The number of gene copies associated with this pathway can vary widely, even among related species. In the work of Pereira and co-workers from 2019 [104], mutants of the model *S.* sp. PCC 6803 cyanobacterium was created to observe the effect of deletion of selected genes involved in the wzy-dependent pathway. Phenotypic changes were not observed for the ∆*wzy (*∆*sll0737*) and ∆*wzx* (∆*sll5049*) mutants. On this basis, their functional redundancy was suggested. On the other hand, for mutants Δ*wzc* (Δ*sll0923*) and Δ*wzb* (Δ*slr0328*) a change in the amount and composition of EPS was demonstrated. It was found that Wzc is involved in the production of capsular polysaccharides (CPS) and RPS, while the Wzb protein influences the production of RPS only [104]. The Wzc protein was also shown to have ATPase and autokinase activities which is typical for bacterial tyrosine kinases [104]. It has been shown that Wzc phosphorylation is controlled by Wzb phosphatase, which shows that the tyrosine phosphorylation status is crucial in the production of EPS by *S.* sp. PCC 6803 [89,95,97,104,105].

Another mechanism for the EPS synthesis is the ABC transporter-dependent pathway, used e.g., for the capsular polysaccharides (CPS) production [106]. An example of such a polymer is CPS with poly-2-keto-3-deoxyoctulosonic and glycolipid phosphatidyl glycerol acid at the reducing end (produced by thermophilic cyanobacteria *Mastigocladus laminosus)* [95,106], which has anti-cancer properties. Typically CPS is formed with a single operon containing GT, but heteropolymers can also be obtained using ABC-dependent mechanism if multiple GTs are engaged in the assembly process [95,106,107]. The EPS in this pathway is fully polymerized on the inner lamina of the plasma membrane before further transport. The transfer of amplified units in the case of CPS takes place through the ABC transporter, which contains the KpsM and KpsT proteins. It includes the inner membrane and the PCP KpsE and the OPX KpsD proteins [89,107]. CPS formed in this way has a phosphatidylglycerol at the reducing end of the glycolipid phosphatidylglycerol and a linker which is a Kdo (poly-3-deoxy-d-manno-oct-2-ulosonic acid). This linker is synthesized with the KpsC, KpsF, KpsS and KpsU proteins [89]. Transport across the inner membrane and translocation to the cell surface, however, may differ for different polymers [89,101,102]. This feature distinguishes a polymer produced by the Wza dependent pathway from that produced by the ABC transporter dependent pathway. The mechanisms of action of ABC transporters may be distinguished by the presence (or absence) of the characteristic non-reducing terminal modifications of the exported substrates. They serve as chain termination and/or export signals, and the presence (or absence) of a discrete substrate-binding domain at the nucleotide-binding polypeptide of the ABC transporter domain. In the study by Pereira and co-workers from 2019 [104], no phenotypic differences were found for *S.* sp. PCC 6803 ∆*kpsM* (∆*slr2107*) mutants and ∆*kpsM* ∆*wzy* (∆*slr2107* ∆*sll0737*) mutants. The PCP and OPX proteins involved in the export of EPS through the Vasa and ABC-dependent pathways as well as OPX and PCP homologs were identified by Pereira and co-workers for most of the studied cyanobacteria [89,95,97,98,104].

The final pathway discussed here is the synthase dependent pathway, which is a process that is flippase-independent [97]. In this process, EPS is synthesized and also transported across the plasma membrane with the help of a specific synthase. For this synthetase, a cyclic-di-GMP signaling molecule is required [89]. Often homopolymers, requiring only one type of sugar precursor, are formed in this pathway [95,97]. Initially, alginate is synthesized as polymannuronic acid, which is processed by epimerases and further processed to glucuronic or mannuronic acid. The proteins participating in the process of alginate modification in the periplasm are AlgF, AlgG, and AlgL. The last mentioned protein degrades accumulated alginate. Its transport is dependent on the AlgK scaffold protein and the AlgE porin [89,95,97,98].

Another example of an exopolysaccharide synthesized in a synthase-dependent pathway is cellulose, which is a long-chain polymer of β-1,4-d-glucan molecules. One of the most important steps in cellulose synthesis is the activation of the PilZ domain of the cellulose synthase catalytic subunit by cyclic di-GMP [108]. Some cyanobacteria have a single *CesA* gene encoding cellulose synthase, and the effect of its activity have been observed in vivo. Under conditions of limited salinity, *S.* sp. 7002 mutants lacking this gene grew slower than the WT and were deformed. Cyanobacteria can produce a cellulose-containing layer between the outer and cytoplasmic membranes [108,109,110]. Cellulose is also responsible for cell aggregation, especially when its production is induced by light and low temperature [111]. Maeda et al. have shown that in *Thermosynechococcus vulcanus*, cell aggregation through cellulose occurs via two light-induced receptors SesA, SesB, and SesC. They are responsible for the regulation of the cyclic di-GMP cellular level. In the same study, mutant strain with increased expression of the gene encoding Tlr1210, a diguanylate cyclase grew normally only after blue light-and temperature (31 °C) induction [112]. Also, overexpression of tll0007 gene, encoding the cellulose synthase catalytic subunit (XcsA) did not increase cellulose production. However, deletion of this gene blocked cell aggregation [112]. Surprisingly two genes located apart from *xcsA* gene in *T. vulcanus* genome were identified as essential for cellulose-mediated cell aggregation. These genes are: hlyD-like protein *tlr0903* (*xcsB*) and endoglucanase-like *tlr1902* (*xcsC*). Furthermore, a Tol-C like protein encoded by *tlr1605* was also essential for successful cellulose production. The authors propose tripartite secretion system for cellulose production mechanism in this cyanobacterium [112,113]. The level of cellulose production in cyanobacteria is relatively low and not sufficient for commercial use [110]. For this reason, studies have been carried out to increase the expression of native genes or to introduce all genes forming cellulose synthase operon from other genera. Particularly noteworthy is the study in which the genes of the *cmc*, *ccp*, *cesAB*, *cesC*, *cesD*, *bgl* operon were introduced from *Komagataeibacter xylinus* (formerly *Gluconacetobacter xylinus*) into *S.* sp. PCC 7002 [110]. High cellulose production was observed for the mutant with above mentioned operon but lacking the native *cesA* gene. It was also recognized that the cultivation of cyanobacteria in low salinity is important, possibly because cyanobacteria under such conditions produce more cyclic di-GMP [108,109].

Many environmental factors affect the efficiency of EPS production. Optimum growing conditions, however, are often species-specific, and strain-specific differences can even be observed. One of the most important factors is access to light. Constant and high light intensities increased EPS production in *Arthrospira platensis* or *Nostoc* sp. [114,115]. Illumination intensity and the temperature usually have a synergistic effect on biosynthesis. Temperature affects photosynthesis because of its effect on nutrient uptake and oxygen secretion by PSII [116,117]. There are, however, also contradictory studies that show a negative or no effect of temperature increase. Elevated EPS production may also be caused by phosphate starvation or its low level [118,119]. However, as with temperature, some studies show the opposite results [120]. Interestingly, it has been shown by metagenomic analysis for flagellar *Nostoc* cyanobacteria that the spectra of the light used during culturing has an effect on the amount of EPS produced. The red light increased the production of polysaccharides by inducing photoprotection [121]. Carbohydrates accumulate due to the need to use excess electrons from PSII and activate the carboxylic acid cycle. It has been shown that the deficiency of potassium and manganese promotes EPS synthesis. The increase in carbon dioxide concentration negatively affects the accumulation of EPS due to the carbon concentration mechanism (CCM). EPS can be synthesized as a cell’s stress response. This correlates with the information on the protective role of EPS in cell [95,99,122].

Polysaccharides synthesis pathways are at the present level of their understanding the least probable to be successfully engineered in new cyanobacterial strains because enzymatic machineries involved in polysaccharides synthesis and secretion are very complex. Moreover, these pathways are very diverse when cyanobacterial strains are compared. It is a great challenge to obtain polysaccharide-based biomaterial of desired properties, composed of an exactly designed pattern of monomers. From the other point of view, polysaccharide precursors may be used in other biosynthetic pathways. Therefore, limited EPS production, not invading their natural protective role in cellular homeostasis, may be an intriguing prospect.

## 7. Conclusions

Cyanobacteria, photosynthetic microorganisms that utilize light as an energy source and carbon dioxide as a primary carbon source are promising microbial cell factories for sustainable production of many biochemicals including biopolymers. These properties give a promise of sustainable production of essential biopolymers directly from CO_2_, bypassing the need for an intermediate carbohydrate platform and resultant carbon losses associated with respiratory metabolism of heterotrophic microbial cell factories. Three groups of bioproducts are particularly interesting, the primary storage molecule of cyanobacteria, glycogen; extracellular polymeric substances, primarily β-1-4 linked glycans, including cellulose; and polyhydroxyalkanoates, mainly represented by another storage molecule polyhydroxybutyrate. The developments in genetic engineering and synthetic biology of cyanobacteria enable a deeper understanding of their metabolic processes responsible for biopolymer synthesis, dissection of biosynthetic mechanisms and ultimately optimization of their production so they could match the needs of growing biopolymer industry. The advances in understanding the cyanobacterial metabolism revealed that two central metabolic pathways of cyanobacteria, carbon fixing Calvin-Benson-Bassham cycle and tricarboxylic acid cycle are fundamentally connected to the biosynthesis of these molecules. It was revealed that there are important differences between the biosynthesis of these biopolymers in cyanobacteria and heterotrophic organisms. Since light-harvesting and CBB are responsible for energy and carbon supply in cyanobacteria the relationships between biosynthesis of essential polymers is also altered. Photosynthetic production of the primary photosynthetate, glyceraldehyde-3P, is closely connected to central carbon metabolism (EMP, ED, OPP) it has knock-on effects in storage compound metabolism and in the TCA cycle. Glycogen synthesis and breakdown is closely connected with the circadian clock in response to the photoperiod. Simultaneously the carbon flux from the glycogen breakdown is directed towards the OPP and regeneration of RuBP when cells prepare for initiation of photosynthesis in light photoperiod. The close association between the reserve storage material and carbon fixation is arguably the main reason why several approaches that aimed at channeling glycogen carbon flux towards other products were not successful. Future attempts should take into consideration the close relationship between glycogen biosynthesis, circadian clock, and product formation with intermittent carbon rechanneling likely to be more successful than permanent impairment of glycogen metabolism. When it comes to the synthesis of other complex carbohydrates, including EPS, there is still much progress to be made, but their close metabolic proximity to the primary photosynthetates and reserve material supply is promising from a flux balancing point of view. On the other hand, a relatively complex enzymatic biosynthesis and secretion machinery make it challenging to obtain biomaterials of desired properties. Because of the importance of the CBB in cyanobacteria, TCA acts as a secondary energy source. The implications of this are found both in the enzymatic machinery of the TCA cycle, which is both more diverse and different to standard cycle found in heterotrophic organisms. The TCA cycle in cyanobacteria acts primarily as a source of chemical intermediates for amino acid biosynthesis, nitrogen fixation, and secondary reserve material (polyhydroxyalkanoates) biosynthesis. When attempting to engineer the production of these biopolymers, it should be noted that there is a link between glycogen metabolism and biosynthesis of PHAs, and links to the circadian clock are expected since they affect the supply of the precursor molecules. To summarize, although recent findings revealed more detailed information about the relationships between the major cyanobacterial metabolic pathways resulting in biopolymer synthesis, there is still plenty of interesting questions to answer both in the areas of basic and applied research. The combination of traditional genetic engineering efforts with novel methods of genome editing, systems, and synthetic biology, machine and deep learning and real time biopolymer characterization methods could all be leveraged to obtain a better understanding and ultimately better control and quality of synthesized biopolymers. Whilst not economically competitive with heterotrophic microbes yet, the development of new strains; such as fast-growing cyanobacteria or, extremophilic strains capable of industrial CO_2_ utilization; combined with the development of new tools and thorough understanding of cyanobacterial metabolism can result in quicker industrialization and sustainable production of essential biopolymers directly from CO_2_ in the near future.

## Figures and Tables

**Table 1:** Studies on glycogen in genetically modified cyanobacteria.

Species	Modification	Culture Conditions	Change in Glycogen Content	Phenotype	Reference
*S.* sp. PCC 6803	Δ*glgA1-*Δ*glgA2*	BG-11 without nitrogen	Full inhibition of synthesis	Cell division and phycobilisome degradation capacity impairment	[42]
*S.* sp. PCC 6803	Δ*glgA1 or* Δ*glgA1*	BG-11 without nitrogen	No impact	No impact	[42]
*S.* sp. PCC 7002	Δ*glgAI-*Δ*glgAII*	38 °C 1% (*v*/*v*) CO_2_	4,2% WT	280% WT of sucrose 172% WT of glucosylglycerate	[43]
		38 °C 1% (*v*/*v*) CO_2_, hypersaline	4,4% WT	295% WT of sucrose 171% WT of glucosylglycerate	[43]
		38 °C 1% (*v*/*v*) CO_2_, N-limiting	7,2% WT	84% WT of sucrose 60% WT of glucosylglycerate	[43]
	Δ*glgAI*	38 °C 1% (*v*/*v*) CO_2_	65% WT	260% WT of sucrose 112% WT of glucosylglycerate	[43]
		38 °C 1% (*v*/*v*) CO_2_, N-limiting	67% WT	38% WT of sucrose 55% WT of glucosylglycerate	[43]
	Δ*glgAII*	38 °C 1% (*v*/*v*) CO_2_	60% WT	220% WT of sucrose 108% WT of glucosylglycerate	[43]
		38 °C 1% (*v*/*v*) CO_2_, N-limiting	60% WT	26% WT of sucrose 21% WT of glucosylglycerate	[43]
*S.* sp. PCC 7002	Δ*glgAI-*Δ*glgAII*	30 °C	<1% WT	Slight reduction in doubling time	[50]
*S. e.* PCC 7942	Δ*glgA*	0.2 M NaCl	Lowering > 90%	Decrease of oxygen secretion by 50%	[51]
*S.* sp. PCC 6803	Δ*glgC*	-	Not compatible	Not compatible	[42,52,53]
*S.* sp. PCC 7002	Δ*glgC*	-	Not compatible	Not compatible	[44,54]
*S. e.* PCC 7942	Δ*glgC*	-	Not compatible	Not compatible	[35,55,56,57]
*S.* sp. PCC 6803	*glgC*-knockdown by CRISPRi	28 °C 1% (*v*/*v*) nitrogen starvation	Lowering by 75%	Non-chlorosis	[45]
*S. e.* PCC 7942	*glgC*-knockdown by CRISPRi	BG-11 without nitrogen	Lowering by 74.5-95.2%	Non-chlorosis	[46]
*S.* sp. PCC 6803	*glgC*-knockdown by small RNA *regulatory tools*	BG-11, 30 °C, 50 μmol photons m- 2 s- 1	Lowering by 75%	Not reported	[47]
*S.* sp. PCC 6803	*glgC*- riboswitch regulation	Theophylline 0 μM	Lowering by 60%	No change in growth	[49]
		Theophylline 1100 μM	Increase by 300%	Slightly increased growth	[49]
*S. e*. PCC 7942	*glgC*-knockdown by riboswitch	30 °C 150 mM NaCl 1mM IPTG Theophylline 0 μM	Lowering by 90%	Slight decrease in cell growth, decrease in sucrose accumulation	[48]
*S.* sp. PCC 6803	Δ*glgP1-*Δ*glgP2*	Constant light 2% CO_2_25 °C	107% WT	No impact on chlorophyll content	[58]
*S.* sp. PCC 6803	Δ*glgP1-*Δ*glgP2*	In light during 12 h cycles 2% CO_2_ 25 °C	Increase by 236%	Decrease of chlorophyl lcontent	[58]
*S.* sp. PCC 6803	Δ*glgP1-*Δ*glgP2*	In dark during 12 h cycles 2% CO_2_ 25 °C	Increase by 420%	Decrease of chlorophyl lcontent	[58]
*S.* sp. PCC 7002	Δ*glgP*	1% *v*/*v* CO_2_ 38 °C	Not reported	No effect on growth	[59]
*S. e.* PCC 7942	*OE-cscB*, *OE-sps*, *OE-glgC*	30 °C 150 mM NaCl 1 mM IPTG	160% WT	Increase in the amount of sucrose	[48]

**Table 2:** Recent studies with mutant strains of cyanobacteria producing polyhydroxybutyrate (PHB).

Name of Species	Modification	Culture Conditions	% PHA (dry cell weight)	Time [d]	Phenotype	Reference
*S.* sp. PCC 6803	OE *pha AB*	Nitrogen deficiency	26 %	9	Slower growth	[75]
	OE *pha AB*	Nitrogen deficiency + 0.4% acetate	35%		Slower growth	[75]
*S.* sp. PCC 6803	*WT*	Nitrogen deficiency	∼15%	14	90 µg/108 cells of glycogen	[71]
	Δ *glgP1*		17%		90 µg/108 cells of glycogen	[71]
	Δ *glgP2*		2%		125 µg/108 cells of glycogen	[71]
	Δ *glgP1/2*		2%		110 µg/108 cells of glycogen	[71]
	Δ *pfk1/2*		2.6%		110 µg/108 cells of glycogen	[71]
	Δ *gnd*		7%		105 µg/108 cells of glycogen	[71]
	Δ *pfk1*		11%		105 µg/108 cells of glycogen	[71]
	Δ *pfk2*		12,5%		110 µg/108 cells of glycogen	[71]
	Δ *eda*		17%		120 µg/108 cells of glycogen	[71]
	Δ *glgA1*		6%		103 µg/108 cells of glycogen	[71]
	Δ *glgA2*		17%		110 µg/108 of glycogen cells	[71]
	Δ *glgC*		18%		Lack of glycogen	[71]
*S.* sp. PCC 6803	OE *xfpk*	2% CO_2_	5%	25	Accumulation before nitrogen depletion	[76]
	OE *xfpk pta* and *ach* knock out.		12%	32	Accumulation before nitrogen depletion	[76]
*S.* sp. PCC 6803	WT	Light + nitrogen deficiency	12%	13	Not reported	[77]
		Light limitation + nitrogen deficiency	17%			[77]
	Δ zfk	Light + nitrogen deficiency	13%			[77]
		Light limitation + nitrogen deficiency	19%			[77]
	Δ *pfk*	Light + nitrogen deficiency	6%			[77]
		Light limitation + nitrogen deficiency	2%			[77]
*S.* sp. PCC 6803	WT	Nitrogen deficiency	9.5%	7	3% fatty acid content under N deprivation	[78]
	Δ *phaB*		0%		2,8% fatty acid content under N deprivation	[78]
	OV *pfabG* + Δ*phaB*		3%		2,5% fatty acid content under N deprivation	[78]
*S.* sp. PCC 6714	WT	Nitrogen and phosphorus deficiency	6%	7		[79]
	Random mutatons		To 29%		Not compatible	[79]
*S.* sp. PCC 7002	Δ*A0171-PHB*	Light + CO_2_	∼4.5%	P(3HB-*co*-4HV)	Increase of total cell dry weight	[80]

## Abbreviations

ATP	Adenosine 5′-triphosphate
CBB	Calvin-Benson-Bassham cycle
CCM	Carbon concentration mechanism
Chl	Chlorophyll
CPS	Capsular polysaccharides
Cyclic di-GMP	Cyclic diguanylate
DCW	Dry cell weight
EPS	Extracellular polymeric substances
GABA	γ-aminobutyric acid
GT	Glycosyltransferases
LPS	Lipopolysaccharide
NAD+/NADH	Nicotinamide adenine dinucleotide
NADP+/NADPH	Nicotinamide adenine dinucleotide phosphate
NPQ	Non-photochemical quenching
OGDH	2-oxogluterate dehydrogenase
OPP	Oxidative pentose phosphate
OPX	Extra-membrane polysaccharides
PCC	Pasteur Collection of Cyanobacteria
PCP	Polysaccharide copolymerase
PHA	Polyhydroxyalkanoate
PHB	Polyhydroxybutyrate
PSI	Photosystem I
PSII	Photosystem II
RPS	Released polymer substances
TCA	Tricarboxylic acid cycle
WT	Wild type
